# Measurable Residual Disease in Hematological Cancers

**DOI:** 10.3390/cancers16223722

**Published:** 2024-11-05

**Authors:** Krzysztof Jamroziak, Bartosz Puła

**Affiliations:** 1 Department of Hematology, Transplantation and Internal Medicine, Medical University of Warsaw, Banacha 1a, 02-097 Warsaw, Poland; 2 Department of Hematology, Institute of Hematology and Transfusion Medicine, I. Gandhi 14, 02-776 Warsaw, Poland; bpula@ihit.waw.pl

Minimal residual disease (MRD) is most easily defined as a minimal amount of cancer cells that remain following the treatment of the disease, potentially owing to disease recurrence and the patient’s dismal prognosis. In recent years, quantifying this parameter has demonstrated an increased interest in the hematological society, opening further possibilities for personalizing patient treatment and outcomes (1). Precision Healthcare aims to apply classification algorithms to match interventions to underlying illness mechanisms in patient subgroups, improving diagnosis and therapy by stratifying patients. MRD measurement may become its essential part. MRD in hematologic malignancies may be utilized for various clinical concepts ranging from disease relapse monitoring, response assessment, deciding to escalate or end the therapy at specific time points, and predicting disease recurrence [1,2,3]. So far, the best examples of MRD utilization in hematological malignancies are described in chronic myeloid leukemia (CML), acute promyelocytic leukemia (APL), and acute lymphoblastic leukemia (ALL), as these diseases possess distinct molecular traits enabling MRD assessment [1,2,3].

Nevertheless, much has to be performed in this field to implement the concept of MRD and MRD-driven personalized medicine. Currently, several methods of MRD quantification are utilized, ranging from broadly applied multicolor flow cytometry (MFC), quantitative real-time polymerase chain reaction (qPCR), and next-generation sequencing (NGS) to newer methods such as liquid biopsy or mass spectrometry [1,2,3]. All of the methods mentioned above have advantages and drawbacks. Therefore, it should be noted that not all are suitable for all scenarios and should be carefully analyzed in a particular disease and clinical context. In some cases, despite the broad possibilities of MRD assessment, its role has not yet been clearly defined in clinical guidelines, as is the case of chronic lymphocytic leukemia (CLL) and multiple myeloma (MM) [1,2,3,4].

In this Special Issue of Cancers, titled “Measurable Residual Disease in Cancer”, six original research articles address recent trends and challenges in MRD assessment regarding four hematological malignancies: ALL, acute myeloid leukemia (AML), MM, and diffuse large B-cell lymphoma (DLBCL). These papers cover important issues regarding MRD assessment, including the indications and time points of measurement, methodology, and interpretation of results, as well as incorporation into clinical routine. Three papers in this Special Issue are devoted to different issues related to MRD in acute leukemias and what underlies the importance of MRD utilization in these malignancies [1,2].

MRD assessment of ALL has become integral to Philadelphia-positive (ALL-Ph+) and Philadelphia-negative (ALL-Ph-) cases. It is incorporated into numerous treatment protocols and drives therapeutic decisions, especially in the setting of qualifying patients for treatment escalation or allogeneic hematopoietic stem cell transplantation (allo-HSCT). In their paper, Pawinska-Wasikowska et al. report the applicability of MFC in the assessment of early blast clearance and its impact on the outcomes of children with acute lymphoblastic leukemia treated according to the ALL IC-BFM2009 protocol at their center. The rationale for this study was that a single threshold for assigning patients to the MRD risk group only sometimes correlates with the response kinetics. Regardless of the high leukemia burden at the early time points of the assessment, some patients present low or undetectable MRD at later measurement points. Early blast clearance, measured by MFC, allows for the continuous evaluation of leukemic burden in bone marrow, making the kinetics of its clearance also an informative indicator of outcome in childhood ALL. The authors found that the most informative cut-off and time point of MRD measurement in bone marrow during therapy was 0.1% at day 33 (end of induction) for predicting relapse. Patients with negative MRD on days 15 and 33 had a higher 5-year overall survival rate (100%) and a higher relapse-free survival rate (97.6%) than those with positive levels of MRD (≥0.01%) at both time points (77.8% and 55.6%, *p* = 0.002 and 0.001, respectively). Most patients with residual disease below 0.1% on day 15 exhibit hyperdiploidy or ETV6-RUNX1 mutation in malignant cells. The authors, therefore, concluded that early MRD measurement combined with streamlined genetic analysis can help distinguish between individuals at low and high risk, enabling tailored treatment plans and better results for pediatric ALL patients.

The work mentioned above underlies the role of MFC MRD assessment in pediatric ALL patients; however, in the case of adult B-cell ALL (B-ALL) and AML, its utility is increasing, but further studies are needed to standardize this method. Its correlation with molecular data may bring novel prediction models. It should be stressed that in the case of AML patients, molecular assessment is limited to several genes, and approximately 40–50% of AML patients do not have a discriminatory mutation for potential disease monitoring [1,2,5]. In their prospective observational study, van de Linde et al. compared the performance of MFC and molecular MRD assessment methods in adult B-ALL and AML regarding the risk of disease relapse and one-year survival outcomes. Their study confirmed that in the case of AML, the current role of molecular testing is limited to particular subtypes (e.g., *NPM1* mutations and core binding factor translocations) and reflects the disease’s heterogeneity; however, in B-ALL, a moderate correlation between MFC and molecular assessment was disclosed.

Nachmias and colleagues addressed the limited role of molecular MRD assessment in AML. Only NPM1 and core-binding factor-mutated leukemias may be monitored using quantitative polymerase chain reaction (qPCR), correlating with disease burden and patient outcomes. Work is being performed on other mutated AML genes. However, the lack of standardization of the measurements and laboratory heterogeneity limit the use of molecular testing for other AML subtypes. Nachmias et al. created a vector containing a synthetic minigene that contains the *ABL1* gene and the sequences of the desired gene mutation. Then, by including the ABL1 sequence, the copy number of each synthetic minigene may be quantified, enabling matched copy number estimation of the mutation of interest using commercially available ABL1 standards. This method provides an objective and sensitive instrument for molecular MRD evaluation. The proof of concept was tested in 19 patients with atypical *NPM1*, *RUNX1*, and *IDH1/2* mutations. Response and MRD levels were correlated in each case. Most notably, MRD monitoring of atypical NPM1 and RUNX1 mutations detected relapse while the patient was still in complete remission using flow cytometric assessment.

Two other articles in this Special Issue cover the role of MRD in MM. In this disease, MRD has been widely established as a prognostic marker, and currently, several trials address the issue of MRD-driven therapies in relation to disease risk and to minimize treatment adverse events [6,7]. In MM, MRD is most often measured using MFC. However, molecular methods are intensively being investigated, enabling a more detailed and possibly more reliable analysis of leftover cancer cells. In their work, Bors et al. focused on MM patients bearing the translocation (4;14). This molecular abnormality affects approximately 15% of MM cases and results in an IGH::NSD2 fusion transcript. Breakage occurs in three major breakpoint regions within the *NSD2* gene (MB4-1, MB4-2, and MB4-3), which the authors were able to measure using qPCR and digital PCR in peripheral blood and bone marrow in 111 MM patients. A sensitivity of 10^−6^ could be achieved, but it was lower in peripheral blood samples than bone marrow. The authors also confirmed the recent findings that the MB4-2 transcript is associated with patients’ worse survival.

The paper by Jiang et al. describes an attempt to minimize the effect of hemodilution on the MRD MFC measurement in MM patients. In their work, the authors assessed the influence of a series of centrifugations and bone marrow aspirations on the enrichment of preparations and minimization of the occurrence of false negative MRD results. They found that using routine bone marrow samples for assessing MRD levels may overestimate MRD-negativity rates. In contrast, the analysis of enriched samples was more sensitive regarding MRD detection. Therefore, the authors suggest that it may be a practical preanalytical method for flow cytometric MRD detection to increase MRD detection sensitivity.

Finally, the study of Figaredo and colleagues focuses on assessing the potential value of liquid biopsies in diagnosing and monitoring disease in the most common B-cell non-Hodgkin’s lymphomas (B-NHL). Although blood and bone marrow are the most utilized tissue compartments in the case of leukemias, in the case of lymphomas, cancer cells may reside in other hardly accessible tissue compartments, e.g., lymph nodes, spleen, and other extranodal localizations. Liquid biopsies utilize the presence of cell-free DNA in the plasma of lymphoma patients, which increases the ability to detect lymphoma. Potentially implementing liquid biopsies would minimize or render tissue and bone marrow biopsies obsolete [8,9]. However, some limitations still have to be overcome, such as the standardization of sample collection and processing, specificity of the techniques used, and sampling timing to utilize this method routinely in the future. Figaredo et al. performed a multicenter study on 78 patients with B-NHL (25 follicular lymphomas and 53 DLBCL) using NGS of cfDNA liquid biopsies and paired gDNA tissue biopsies at diagnosis, comparing the mutational statuses with PET-CT scan assessment. The authors identified mutations in 71% of liquid and 95% of tissue biopsies and found a correlation between variant allele frequency of somatic mutations. Additionally, mutations in 73% of liquid biopsies from patients with no mutations detected in tissue biopsy or no available tissue samples were identified. Liquid biopsy MRD assessment had a lower sensitivity but a higher specificity than PET-CT. Therefore, a combined evaluation of treatment response using both techniques would provide more accurate and reliable information regarding patient prognosis.

In conclusion, MRD has become a key aspect of disease monitoring in acute leukemias and CML, and its importance is growing as its use expands to lymphoid malignancies with the introduction of new techniques. This method still has some limitations, and a strong dependence on disease heterogeneity is observed. Based on numerous studies, it can be concluded that the possibility of combining liquid biopsy with NGS may soon result in new guidelines for disease diagnosis and treatment monitoring. High-quality randomized clinical trials with well-defined MRD-based endpoints are necessary to implement this methodology on a large scale.
